# Babinski-Nageotte Syndrome Diagnosed in Postpartum Period

**DOI:** 10.1155/2016/5206430

**Published:** 2016-02-16

**Authors:** Serdar Oruç, Hayri Demirbaş, Abdullah Güzel, Mehtap Beker Acay, Mehmet Yaman

**Affiliations:** ^1^Department of Neurology, Afyon Kocatepe University School of Medicine, 03200 Afyonkarahisar, Turkey; ^2^Department of Radiology, Afyon Kocatepe University School of Medicine, 03200 Afyonkarahisar, Turkey

## Abstract

Babinski-Nageotte Syndrome (BNS) is one of the brainstem syndromes characterized by muscle weakness in the opposite half of the body with classic Wallenberg findings. According to our literature survey, only a few cases have been reported and none of them was in the postpartum period. We report a case of a typical BNS in a postpartum woman with an ischemic lesion in the medulla oblongata shown on magnetic resonance imaging.

## 1. Introduction

Firstly diagnosed in 1902, Babinski-Nageotte Syndrome (BNS) is one of the brainstem syndromes characterized by medulla oblongata ischemia [1]. In BNS, which has been described in very few cases in the literature, lateral medullary syndrome symptoms as well as contralateral motor deficit have been observed. Here, we have reported a case diagnosed with BNS which was developed on the 10th day of the postpartum period.

## 2. Case Report

**Figure 1:**
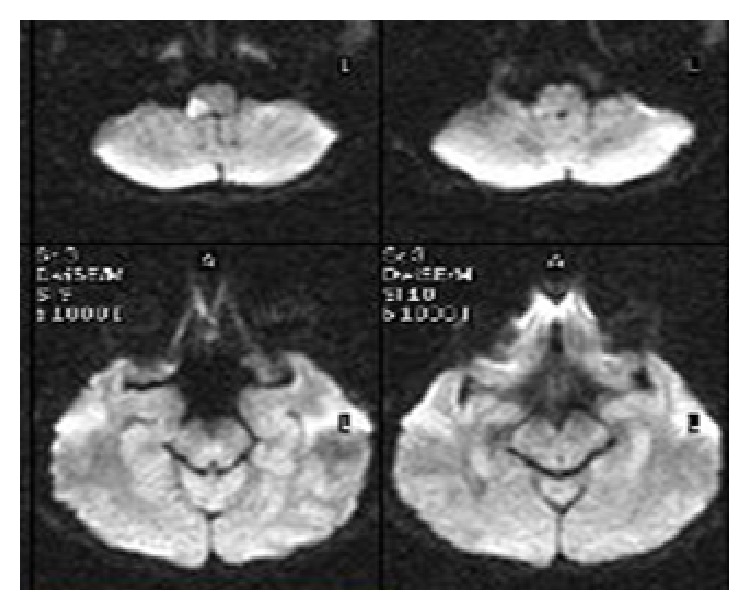
The diffusion restriction which led to the acute infarct, holding the posterolateral bulbus right hemisphere according to the results of the diffusion MRI.

**Figure 2:**
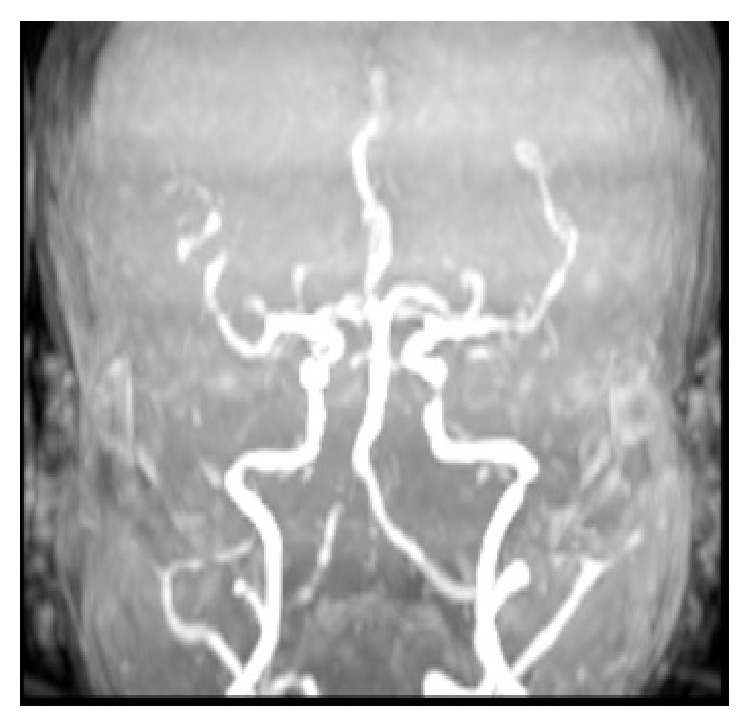
Stenotic segment determined in the right vertebral artery distal according to the MR angiography.

A 30-year-old woman was evaluated with complaints of dysarthria, dysphagia, dizziness, nausea, vomiting, and weakness of left arm and leg. According to her medical history, there was sudden development of dizziness, nausea, and vomiting symptoms one hour before her admission to the hospital and later complaints of dysphagia, dysarthria, and weakness of left arm and leg were added to her presenting complaints. There was no background or family history of the patient with the exception that she had given birth by Caesarean section, 10 days before, and was diagnosed with preeclampsia in the 33rd week of her pregnancy. In her first evaluation, the patient was in stuporous state and she had dysarthric speech. During examination of her eye movements, vertical and horizontal nystagmus were observed. There was flattening of left sided nasolabial sulcus with abnormal gag reflex observed during cranial nerve examination. In the motor system examination, the left upper and lower extremity muscle power were 3/5 level and her Babinski reflex was found to be an extensor response on the left side. During the sensory system examination, pain and thermal senses of the patient were decreased on the left side of the body and cerebellar tests were abnormal on the right side. Evaluation of the cranial MRI screening of the patient with the misdoubt of cerebrovascular disease showed results that were consistent with diffusion restriction which was thought to be acute infarct extending to inferior cerebellar peduncle with involvement of right sided posterolateral medulla oblongata (Figure 1). In magnetic resonance angiography (MRA), stenosis was seen in the distal segment of right vertebral artery (Figure 2).

In addition to lateral medullary syndrome symptoms, there was also contralateral muscle weakness observed in the patient. Therefore, we reached a conclusion that the patient had BNS and medical treatment was initiated accordingly. The patient was hospitalized for 15 days in our clinic. At the end of the fifteenth day her neurological symptoms improved except ataxia and dysarthria.

## 3. Discussion

Babinski-Nageotte Syndrome (BNS) is one of the brainstem syndromes characterized by muscle weakness in the opposite half of the body with accompanying classic Wallenberg findings. Of the vertebral artery branches, anterior spinal artery and posterior inferior cerebellar artery could be observed to be affected together [2]. Despite the developments in recent years in imaging methods, it has been observed that it has been still rarely reported [3]. Particularly, there are some contradictive reports related to its difference from hemimedullary syndrome. However, these contradictions have been prevented in recently performed studies; the topographical and clinical differences between BNS and hemimedullary syndrome have been shown [4]. Lateral and medial medullar regions are involved in medulla oblongata in hemimedullary syndrome whereas the involvement is limited to lateral medullar zone and corticospinal tract in BNS. The hypoglossal palsy is one of the major differences in the differential diagnosis of hemimedullary syndrome and BNS [4].

In the etiological studies of the cases presented in the literature, atherosclerotic occlusions of the vertebral artery as well as pathologies such as syphilitic endarteritis have been identified. However, the most common etiological reason is the vertebral artery dissection [3]. In our case, since there is no risk factor for atherosclerosis and rapid regression in neurologic deficits, it was thought to be the vertebral artery dissection. Our hypothesis is supported since our patient is in the postpartum period and dissections are commonly observed in this period [5]. There is no BNS case reported in the literature during the postpartum period. Here, we have rare case of BNS seen in postpartum period and we believe that it is beneficial to report this case.
